# Supplementary material for: Paracrine effect of regulatory T cells promotes cardiomyocyte proliferation during pregnancy and after myocardial infarction
Source: Nat Commun. 2018 Jun 26;9:2432. doi: 10.1038/s41467-018-04908-z (PMC6018668; doi:10.1038/s41467-018-04908-z)
Supplement: Supplementary file 1 — Supplementary Information [file 41467_2018_4908_MOESM1_ESM.pdf]

Paracrine effect of regulatory T cells promotes cardiomyocyte proliferation during pregnancy and after myocardial infarction

Zacchigna et al.

Supplementary Data

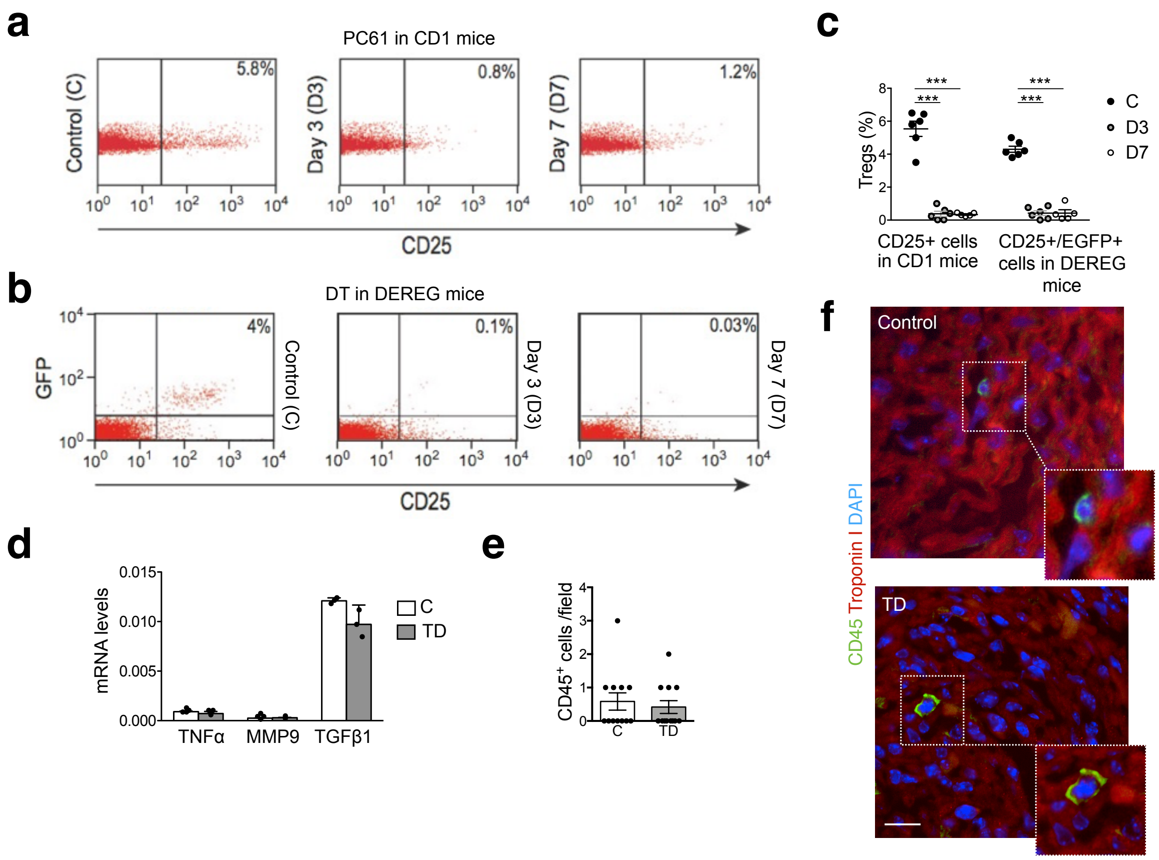

Supplementary Figure 1. a. Flow cytometry plots showing the number of CD25+ Treg cells in the lymph nodes of control CD1 mice (C) and in mice injected with anti-CD25 antibodies (PC61) at day 3 and day 7 after antibody injection. **b.** Flow cytometry plots showing the number of GFP+CD25+ Treg cells in the lymph nodes of control DEREG mice (C) and in mice injected with dyphteria toxin (DT) at day 3 and day 7 after toxin injection. **c.** Quantification of the number of CD25+ Tregs in CD1 mice and GFP+CD25+ Tregs in DEREG mice in control condition or at days 3 and 7 after injection of the depleting agent. **d.** Real-time PCR quantification of the expression levels of TNFα, MMP9 and TGF-β1 in the heart of embryos harvested from pregnant DEREG mothers, either untreated (C) or Treg-depleted by DT injection (TD). Expression of three additional inflammatory genes, IL-6, IFNγ and IL12p40, was tested but not detected in any heart. No statistically significant difference was detected between the two groups for any of the analyzed genes. **e.** Quantification of CD45+ leukocytes in the heart of embryos harvested from pregnant DEREG mothers, either untreated (C) or Treg-depleted by DT injection (TD). **f.** Representative images of the heart of embryos harvested from pregnant DEREG mothers, either untreated (control) or Treg-depleted by DT injection (TD), stained for CD45 (green) and Troponin I (red). Insets shows a higher magnification of the area defined by the white dotted line. Nuclei are counterstained with DAPI. Scale bar, 50 µm. All values are mean ± s.e.m., each dot indicates a biological replicate. One-way analysis of variance and Bonferroni/Dunn's post hoc tests were used to compare multiple groups. ***P<0.001, relative to control.

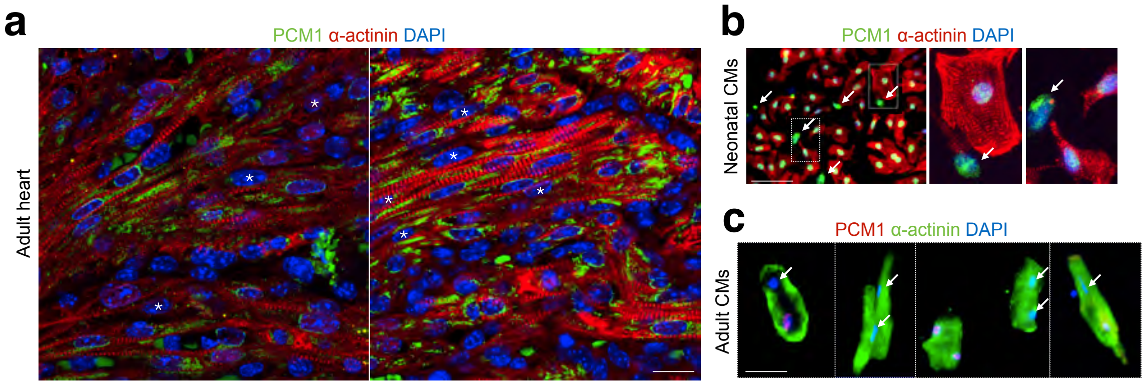

Supplementary Figure 2. a. Sections of a neonatal mouse heart stained for PCM-1 (green) and α-actinin (red). Nuclei are counterstained with DAPI. Asterisks show a few nuclei surrounded by α-actinin staining and having the elongated shape typical of CM nuclei but negative for PCM1 perinuclear staining. Scale bar, 10 µm **b.** Rat neonatal CMs stained for PCM-1 (green) and α-actinin (red). Nuclei are counterstained with DAPI. Arrows indicate PCM1 nuclear staining in cells, which are negative for α-actinin and reasonably represent contaminating fibroblasts. Panels on the right show high magnification images of the details indicated by the white dotted squares. Scale bar, 100 µm. **c.** Adult mouse cardiomyocytes stained for PCM-1 (red) and α-actinin (green). Nuclei are counterstained with DAPI. Arrows indicate PCM1- nuclei inside α-actinin+ CMs. Cells in panels b and c were stained the day after plating. Scale bar, 100 µm. All values are mean ± s.e.m., n=5 biological replicates.

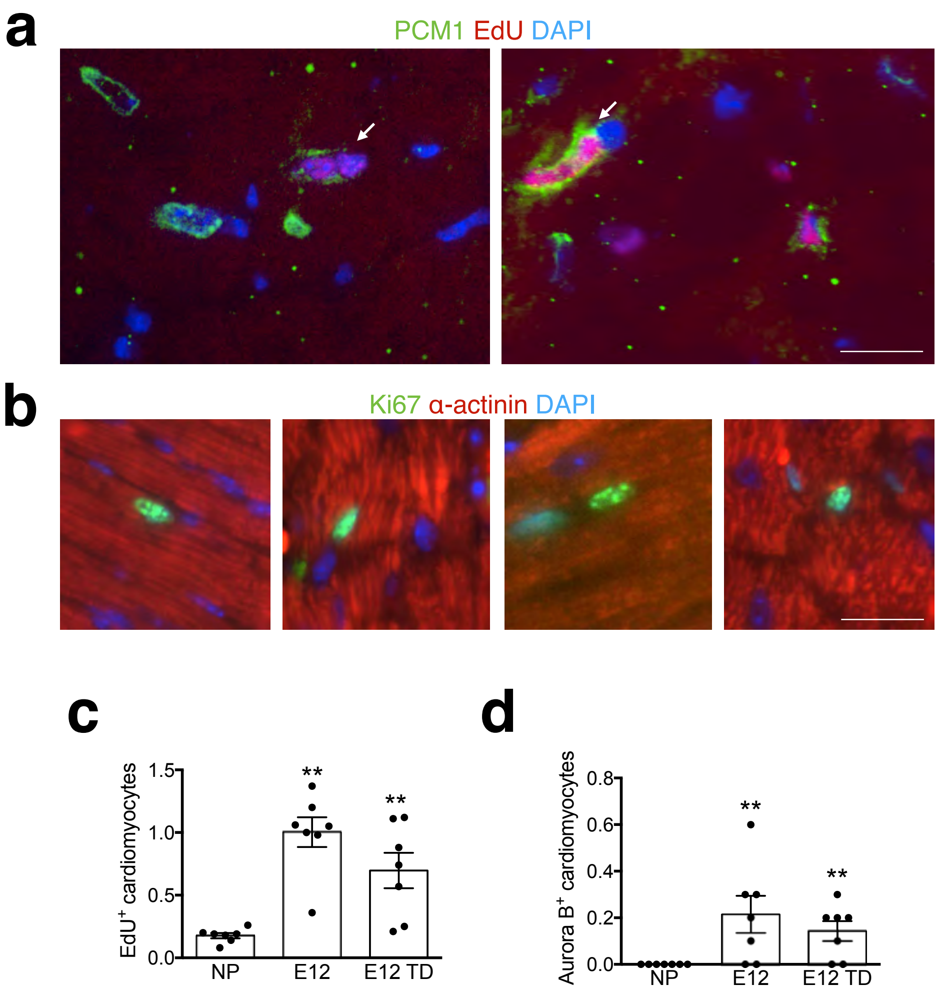

Supplementary Figure 3. a. Sections of the heart of a pregnant mouse stained for PCM-1 (green) and EdU (red). Nuclei are counterstained with DAPI. Arrows indicate nuclei positive for both PCM1 and EdU staining. Scale bar, 25 µm. **b.** Sections of the heart of a pregnant mouse stained for α-actinin (red) and Ki67 (green). Nuclei are counterstained with DAPI. Scale bar, 25 µm. **c.** Quantification of EdU incorporation (% of total CM nuclei) in α-actinin+ CMs of not pregnant (NP), pregnant and Treg-depleted pregnant mice at E12. **d.** Quantification of AuroraB localization at midbodies in α-actinin+ CMs of not pregnant (NP), pregnant and Treg-depleted pregnant mice at E12. All values are mean±s.e.m, each dot indicates a biological replicate. **P<0.05 relative to NP animals.

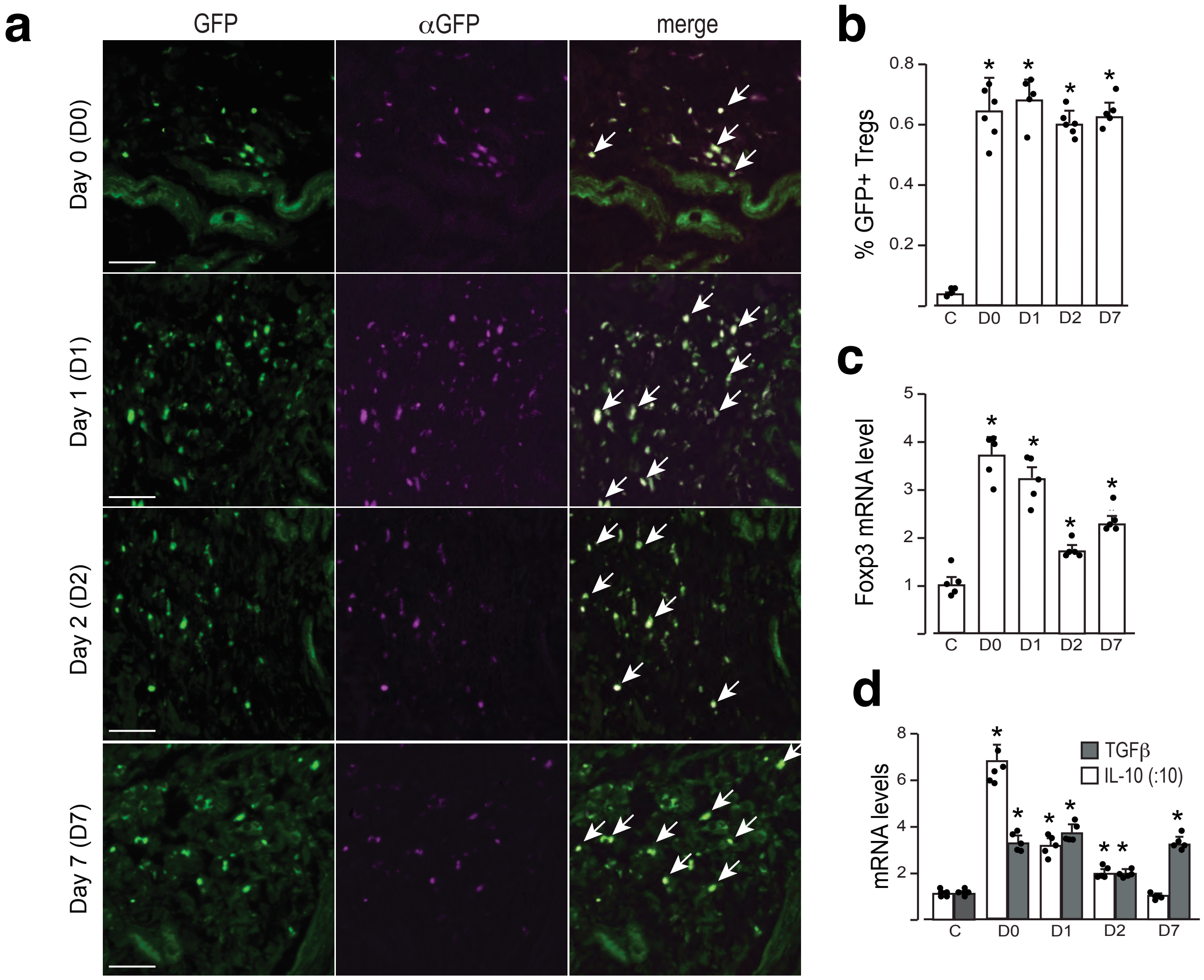

Supplementary Figure 4. a. Heart sections of DEREG mice showing recruitment of GFP+ Tregs at day 0, 1, 2 and 7 after permanent coronary artery ligation. Tregs are identified as GFP+ green cells by fluorescence microscopy and upon labeling with anti-GFP antibodies (stained purple). White arrows indicate Tregs scoring positive in both channels. Scale bar, 100 μm. **b.** Quantification of the number of Tregs in the border region of the infarct (% of total nuclei per microscopic field) at day 0, 1, 2 and 7 after coronary artery ligation. **c-d**. Real-time PCR quantification of the expression levels of Foxp3 (**c**), TGF-β (grey bars in **d**) and IL-10 (white bars in **d**) in control, sham-operated non-infarcted animals (C) and at day 0, 1, 2 and 7 after myocardial infarction. Values are normalized for GAPDH and expressed as fold over untreated (n = 4). All values are mean ± s.e.m., each dot indicates a biological replicate. Pairwise comparison was performed with the Student's t-test. *P<0.05 relative to control.

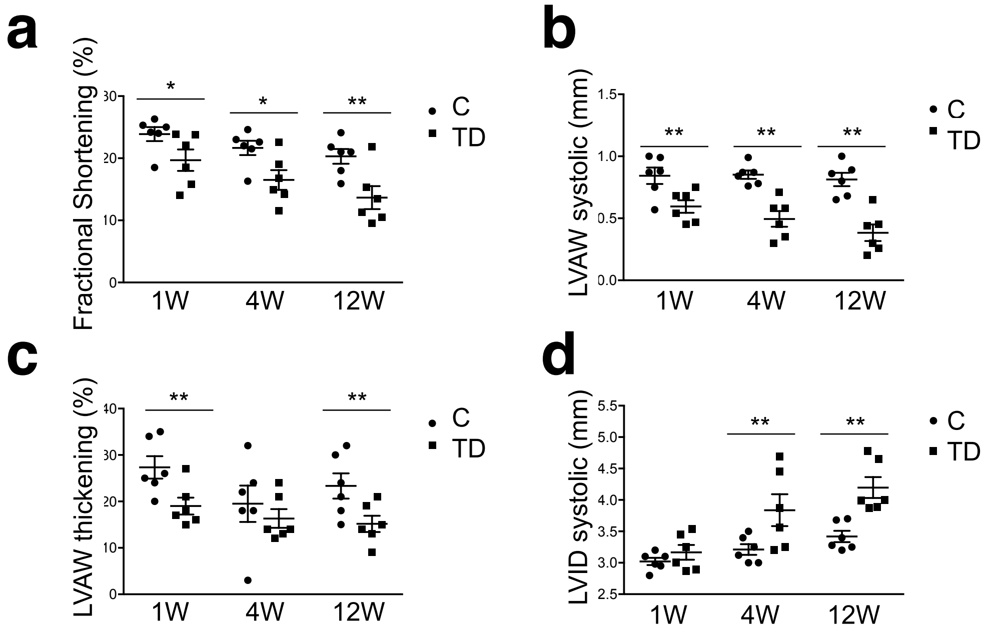

Supplementary Figure 5. Additional parameters of US imaging analysis of cardiac function, including fractional shortening (**a**), left ventricular anterior wall (LVAW) systolic thickness (**b**), LVAW thickening (**c**) and left ventricular internal diameter (LVID) in systole (**d**), in control (C) and Treg-depleted (TD) mice at 1, 4 and 12 weeks (W) after myocardial infarction. All values are mean ± s.e.m, each dot indicates a biological replicate. Two-way ANOVA for repeated measurements was used in d. *P<0.05, **P<0.01, relative to control.

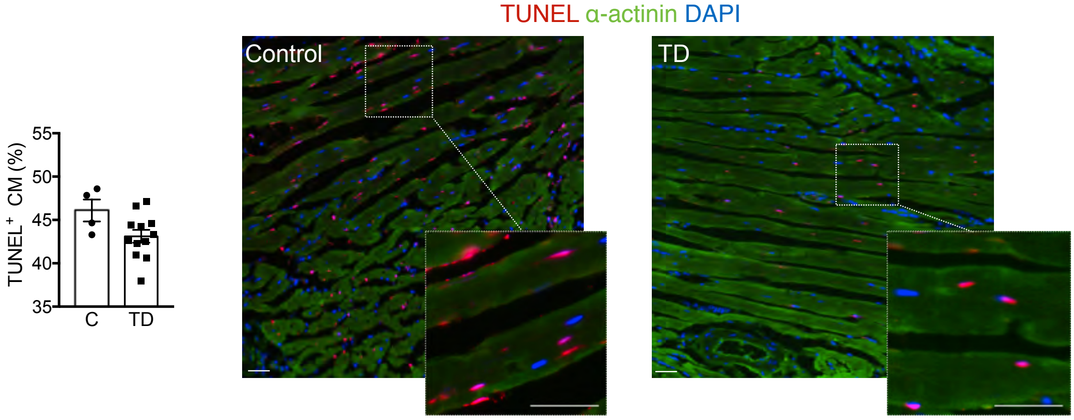

Supplementary Figure 6. Analysis of apoptosis by TUNEL. Quantification and representative images of TUNEL-positive nuclei in the heart of control and Treg-depleted animals. Apoptotic nuclei are stained in red and CMs in green using anti-α-actinin antibodies. Nuclei are counterstained with DAPI. Scale bar, 100 mm. No statistically significant difference was detected between the two groups. All values are mean ± s.e.m, each dot indicates a biological replicate.

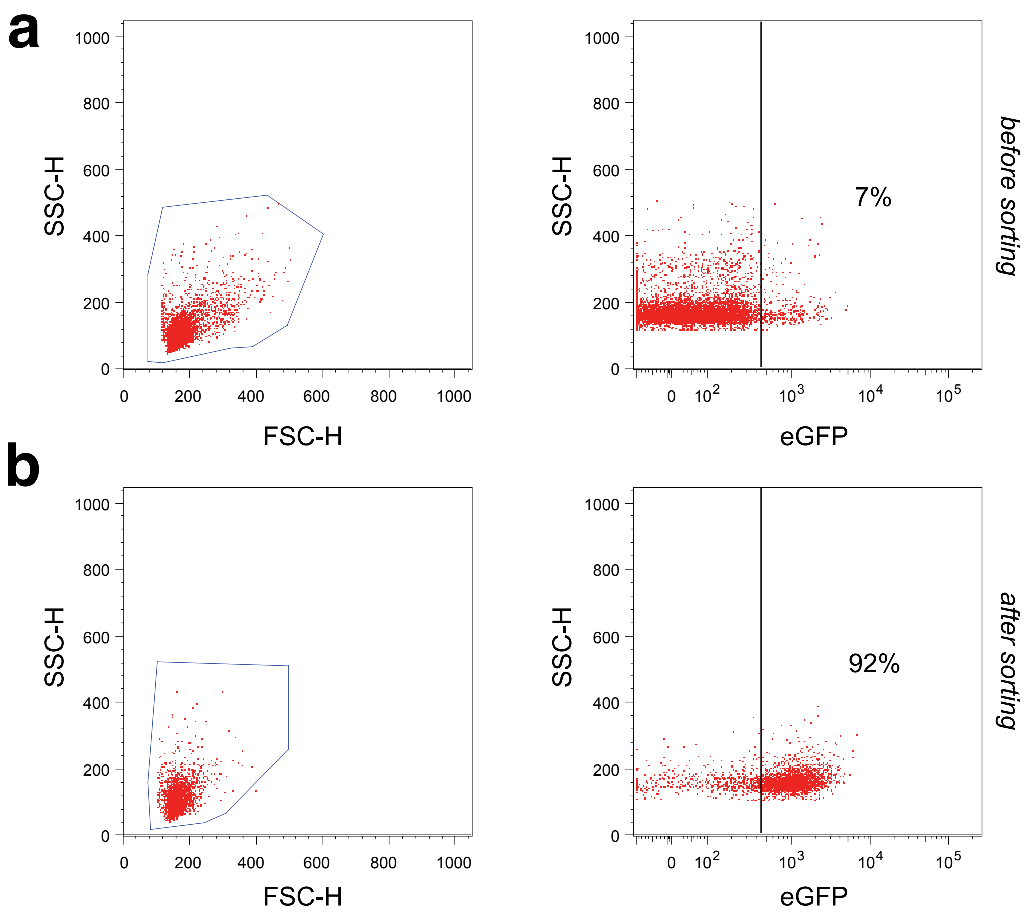

Supplementary Figure 7. Flow cytometry plots showing forward scatter (FSC), side scatter (SSC), and eGFP fluorescence in cells isolated from lymph nodes before (**a**) and after (**b**) sorting of the eGFP+ cells, showing 92% of purity of the sorted cell population.

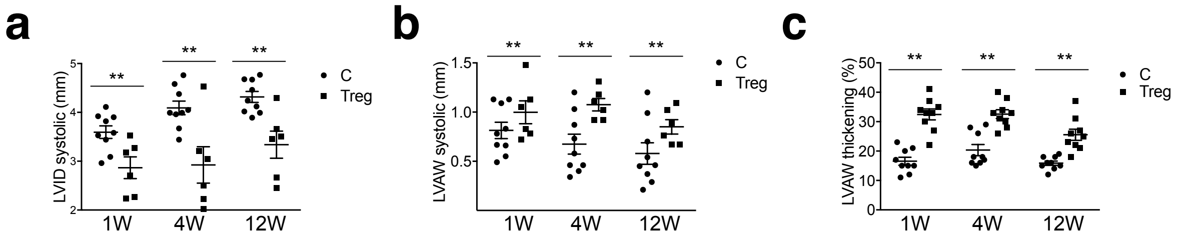

Supplementary Figure 8. Additional parameters of US imaging analysis of cardiac function, including left ventricular internal diameter (LVID) in systole (**a**), left ventricular anterior wall (LVAW) systolic thickness (**b**) and LVAW thickening (**c**) in control (C, circles) and Treg-injected (Treg, square) mice at 1, 4 and 12 weeks (W) after myocardial infarction. All values are mean ± s.e.m., each dot indicates a biological replicate. Two-way ANOVA for repeated measurements was used to follow the same animals over time. **P<0.01, relative to control.

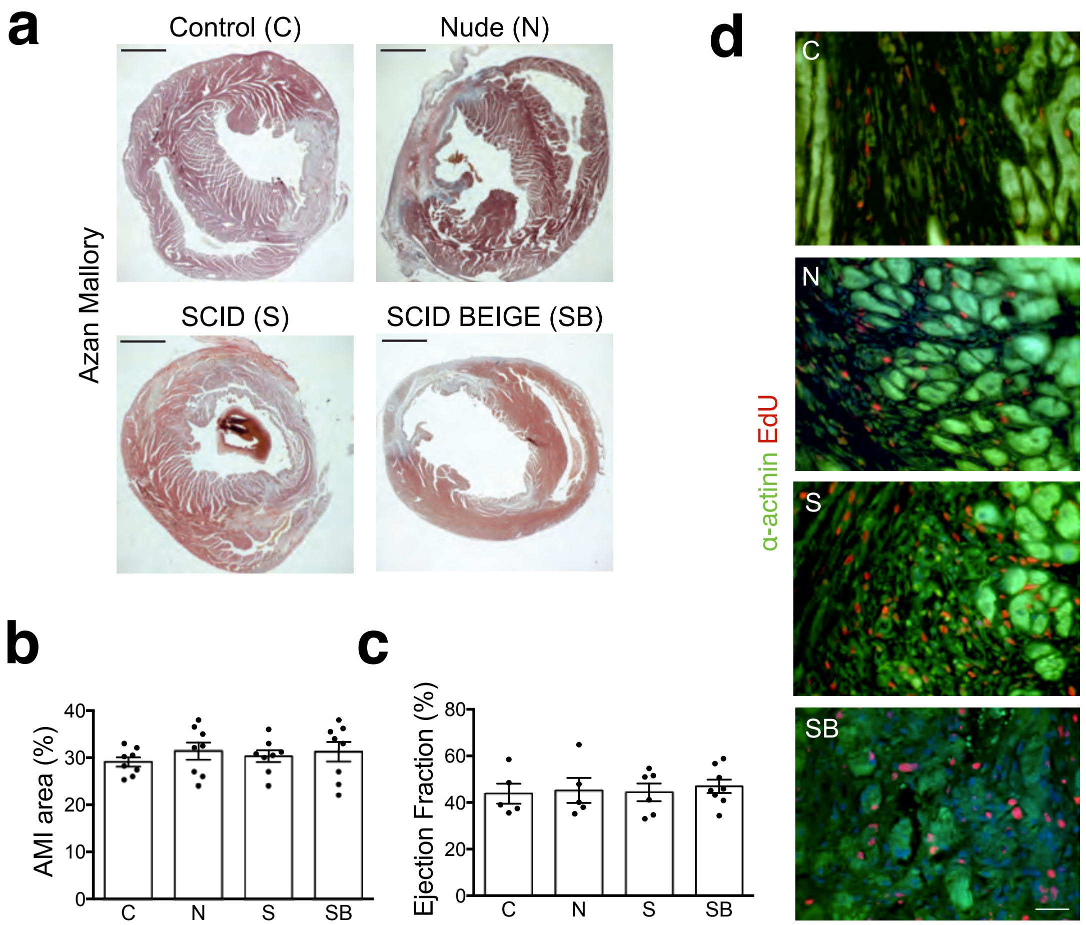

Supplementary Figure 9. a. Representative images of whole transverse sections after Azan Trichromic staining of hearts of wild-type control (C), athymic Nude-Foxn1 (N), Fox Chase SCID (S), and Fox Chase SCID BEIGE (SB) mice at 1 month after myocardial infarction. Fibrotic areas are stained in gray/blue. Scale bar, 1 mm **b.** Quantification of acute myocardial infarction (AMI) area expressed as percentage of the left ventricular area in control (C), athymic Nude-Foxn1 (N), Fox Chase SCID (S), and Fox Chase SCID BEIGE (SB) mice at 1 month after myocardial infarction. **c.** US imaging analysis of the Ejection Fraction of hearts of control (C), athymic Nude-Foxn1 (N), Fox Chase SCID (S), and Fox Chase SCID BEIGE (SB) mice at 1 month after myocardial infarction. **d.** Sections of the heart of control (C), athymic Nude-Foxn1 (N), Fox Chase SCID (S), and Fox Chase SCID BEIGE (SB) mice at 1 month after myocardial infarction, showing EdU incorporation (stained red) by non CM cells (CMs are stained green by anti-a-actinin antibodies). Scale bar, 100 µm. All values are mean ± s.e.m, each dot indicates a biological replicate. One-way analysis of variance and Bonferroni/Dunn's post hoc tests were used to compare multiple groups.

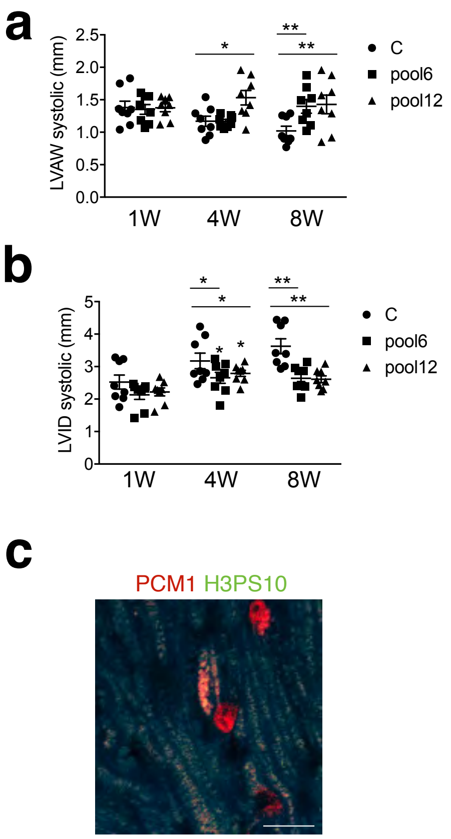

Supplementary Figure 10. Additional parameters of US imaging analysis of cardiac function, left ventricular anterior wall (LVAW) systolic thickness (**a**) and left ventricular internal diameter (LVID) in systole (**b**) in control (C) and AAV-Pool6- and AAV-Pool12-injected mice at 1, 4 and 8 weeks (W) after myocardial infarction. All values are mean±s.e.m. Two-way ANOVA for repeated measurements was used to follow the same animals over time. *P<0.05, **P<0.01, relative to control. **c.** Section of the heart of mice injected with AAV-Pool12, showing a CM nucleus (stained red with anti-PCM1 antibodies) positive for histone H3 phosphorylated on serine10 (H3PS10, stained green). Scale bar, 50 µm. All values are mean ± s.e.m, each dot indicates a biological replicate.
